# Is there a correlation between late re-exploration after cardiac surgery and removal of epicardial pacemaker wires?

**DOI:** 10.1186/s13019-017-0569-5

**Published:** 2017-01-25

**Authors:** Ioannis Bougioukas, Ahmad Fawad Jebran, Marius Grossmann, Martin Friedrich, Theodor Tirilomis, Friedrich A. Schoendube, Bernhard Christoph Danner

**Affiliations:** Department of Thoracic and Cardiovascular Surgery, University Medical Center, Robert-Koch 40 Str, 37075 Goettingen, Germany

**Keywords:** Cardiac surgery, Epicardial pacemaker wires, Pericardial tamponade, Re-exploration

## Abstract

**Background:**

Re-exploration for bleeding accounts for increased morbidity and mortality after major cardiac operations. The use of temporary epicardial pacemaker wires is a common procedure at many departments. The removal of these wires postoperatively can potentially lead to a serious bleeding necessitating intervention.

**Methods:**

From Jan 2011 till Dec 2015 a total of 4244 major cardiac procedures were carried out at our department. We used temporary epicardial pacemaker wires in all cases. We collected all re-explorations for bleeding and pericardial tamponade from our surgical database and then we focused on the late re-explorations, meaning on the 4^th^ postoperative day and thereafter, trying to identify the removal of the temporary pacemaker wires as the definite cause of bleeding. Patients’ records and medication were examined.

**Results:**

Thirty-nine late re-explorations for bleeding, consisting of repeat sternotomies, thoracotomies and subxiphoid pericardial drainages, were gathered. Eight patients had an acute bleeding incidence after removal of the temporary wires (0.18%). In four of these patients, a pericardial drainage was inserted, whereas the remaining patients were re-explorated through a repeat sternotomy. Two patients died of the acute pericardial tamponade, three had a blood transfusion and one had a wound infection. Seven out of eight patients were either on dual antiplatelet therapy or on combination of aspirin and vitamin K antagonist.

**Conclusions:**

A need for re-exploration due to removal of the temporary pacemaker wires is a very rare complication, which however increases morbidity and mortality. Adjustment of the postoperative anticoagulation therapy at the time of removal of the wires could further minimize or even prevent this serious complication.

## Background

The use of temporary epicardial pacemaker wires (EPW) after cardiac procedures is a standard routine at many cardiac surgical departments [1]. These temporary wires are removed postoperatively after a stable cardiac rhythm has been established. A rare but potentially hazardous complication of removal of these wires is the development of a cardiac tamponade necessitating the re-exploration of the patient [1–3]. The exact incidence of this complication remains uncertain, whereas its prevention in the era of dual antiplatelet therapy (DAPT) and modern anticoagulant medicaments, such as novel oral anticoagulants (NOACs), seems to be challenging.

The time point of removal of these EPW is generally dependent upon surgeon’s preference. At our institution, the use of temporary epicardial pacemaker wires is a standard and these are routinely removed on the 4^th^ to 5^th^ postoperative day (POD) by patients in stable cardiac rhythms, whereas in cases of transient atrioventricular block or new postoperative atrial fibrillation demanding electrical or pharmaceutical cardioversion the wires are taken out later. Although careful monitoring of coagulation by means of laboratory measurement of the international normalized ratio (INR) and the activated partial thromboplastin time (aPTT) is standard before removing of the wires, cases of pericardial bleeding are coming up sporadically.

The aim of our work was to gather all confirmed and suspicious cases of pericardial tamponade after removal of EPW and to estimate the frequency of this complication. We also studied the patient’s medications, in order to find a possible correlation, trying to avoid or minimize this risk.

## Methods

From Jan 2011 till Dec 2015, a total of 4244 major cardiac operations have been performed at our department. Paediatric surgical procedures have been excluded from our review.

We collected all reoperations for bleeding and pericardial draining after cardiac surgery in this time period and then we focused on the late procedures, meaning on the 4^th^ POD and thereafter, in order to coincide with the removal of the EPW. As a late re-exploration, we defined a full repeat sternotomy, thoracotomy, thoracoscopy and subxiphoid pericardial drainage insertion.

**Figure 1:**
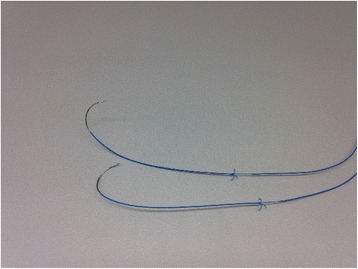
The epicardial pacemaker wires used at our department

Data were obtained retrospectively from our cardiothoracic surgical database. All major cardiac procedures including coronary artery bypass grafting (CABG), aortic, mitral and tricuspid valve surgery through full or partial sternotomy or lateral thoracotomy, surgery of the aorta, transapical aortic valve replacement and heart transplantation were reviewed. At our department, both atrial and ventricular EPW are standard employed to all patients, except by presence of chronic atrial fibrillation, where only ventricular wires are being used. The atrial leads are most commonly sutured on the right atrial appendage, whereas the ventricular leads are sutured either on the ventral or on the diaphragmatic surface of the right ventricle. The anchoring of the ventricular wires requires slightly deeper bites on myocardium in comparison to the atrial ones. At our department, a single type of EPW (TME Tines, Osypka AG, Rheinfelden-Herten, Germany) is being used (Fig 1).

Variables used in our database for searching were bleeding, mediastinal bleeding, reoperation for bleeding, pericardial tamponade, haematothorax and haematoma evacuation either through resternotomy or thoracotomy/thoracoscopy, and subxiphoid pericardial drainage insertion. Reoperations for bypass revision, wound healing disorder or sternal wound infection, secondary closure of a sternotomy, extreme critical procedures with mechanical support of the heart postcardiotomy and chest left open were excluded from this review.

## Results

This 5-year review involved a sample of 4244 patients. A late re-exploration was necessary in 39 patients (0.92%), varying from the 4^th^ till the 30^th^ POD. These late procedures accounted for 17.4% of all re-explorations for bleeding (39 out of a total of 223 re-explorations). Concerning only late re-explorations, our data showed that resternotomy was performed in 20 cases, thoracotomy in five, and subxiphoid pericardial drainage insertion in 14 cases. The primary cardiac operations by these late re-explorations are depicted in Table 1.

**Table 1:** Primary cardiac operations by all late re-explorations for pericardial tamponade (*n* = 39)

Type of operation (*n* = 28)	Number
CABG	5
CABG + MV repair/replacement	3
CABG + AV repair/replacement	10
AVR	7
Myectomy	2
Left ventricular assist device	1
Heart transplantation	1
MV repair/replacement	5
AVR + MV/TV repair/replacement	1
AVR + MV replacement + CABG	2
TAVR transapical	2

(*CABG* = coronary artery bypass grafting, *MV* = mitral valve, *AVR* = aortic valve replacement, *AV* = aortic valve, *TV* = tricuspid valve, *TAVR* = transcatheter aortic valve replacement)

Defining the exact cause of bleeding in the late re-explorations seemed to be complicated, as in many cases no active bleeding could be found. The removal of the epicardial pacemaker wires could be recognized as the definite cause of bleeding in only 8 cases of the total of 4244 patients (0.2%), accounting for 3.6% of all re-explorations for bleeding.

The analysis of the data of these 8 cases showed that, two of the patients had primarily a CABG, three had a valve repair/replacement, one had an extended myectomy and the last two had a combined procedure. All of the 8 patients had an echocardiographic study before removal of EPW showing no pericardial effusion, whereas the clinical findings after EPW removal strengthened the suspicion of a pericardial tamponade. In two cases, the clinical deterioration was so intense that the patients were directly transferred to the operating theater without waiting for an echocardiographic diagnosis. The outcome of these two patients was fatal, whereas the intraoperative findings confirmed an acute pericardial haemorrhagic effusion. In six patients, echocardiography was first performed and then the haemorrhagic effusion was managed either by repeat sternotomy (2 patients) or by insertion of a pericardial drainage (4 patients). In two out of eight patients, an active bleeding on the right ventricle needed to be over sewn. None of these eight patients had a chest tube at the time of EPW removal. Finally, almost all of these patients had a late removal of their EPW (7^th^ till 16^th^ POD) due to need for postoperative pacing (Table 2).

**Table 2:** List of re-explorations for bleeding after EPW removal

Operation	Medication	EPW removal	Reexploration	INR/PTT	Complications
AVR	ASA + VKA + Hep.	16 POD	Pericard drainage	INR = 1.3 PTT = 99	none
AVR	ASA + VKA + Hep.	6 POD	Repeat sternotomy	INR = 1.9 PTT = 41	Blood transfusion (RBC 2 packages)
AVR	ASA + VKA	7 POD	Repeat sternotomy	INR = 2.2	fatal
CABG	DAPT + LMWH	11 POD	Pericard drainage	-	Blood transfusion (RBC 2 packages)
CABG	DAPT + LMWH	9 POD	Repeat sternotomy	INR = 1.3 PTT = 30	Blood transfusion (RBC 2 packages) Wound complications
Myectomy	LMWH	11 POD	Pericard drainage	INR = 1.2 PTT = 28	none
MV repair, occlusion LAA	DAPT + VKA + Hep.	7 POD	Repeat sternotomy	INR = 2.0 PTT = 38	fatal
AVR + CABG + aorta asc. replac.	ASA + VKA + Hep.	12 POD	Pericard drainage	INR = 1.8 PTT = 32	none

(*AVR* = aortic valve replacement, *CABG* = coronary artery bypass grafting, *MV* = mitral valve, *LAA* = left atrial appendage, *asc* = ascending, *replac* = replacement, *ASA* = aspirin, *VKA* = vitamin K antagonist, *Hep* = heparin, *LMWH* = low molecular weight heparin, *DAPT* = dual antiplatelet therapy, *POD* = postoperative day, *RBC* = red blood cells)

The analysis of patients’ medication revealed that, two patients were on DAPT with aspirin and clopidogrel and low molecular weight heparin (LMWH), four were on combination of aspirin with phenprocoumon and heparin as bridging, one patient was on DAPT and vitamin K antagonist (VKA) and one on LMWH as antithrombotic prophylaxis. The INR of patients on phenprocoumon lied between 1.2 and 2.2, whereas aPTT control by patients who were on unfractionated heparin, as bridging till attainment of the optimal INR, was normal by the time of EPW removal with one exception (aPTT > 60). Three from these eight patients needed a blood transfusion due to bleeding, whereas a sternal wound infection after resternotomy in one patient led to a latissimus dorsi plasty (Table 2).

Additionally, our data revealed six highly suspicious cases, which were readmitted in our institution after the 15^th^ POD while on rehabilitation, due to massive pericardial effusion and clinical deterioration. Three of these patients were reexplored through resternotomy and three received a subxiphoid pericardial drainage. All of these patients were discharged after the primary cardiac procedure without an echocardiographic finding of a pericardial effusion and all patients were on VKA and/or aspirin. In three patients, a derailment of anticoagulation (INR >5) was verified. In five patients, the pericardial effusion was older haemorrhagic and in one case it was serous, without blood elements. The outcome of these patients was unproblematic, with the exception of one patient, who developed superficial wound healing complications, demanding a slightly prolonged hospital stay. It remains of course uncertain whether these cases of late pericardial tamponade could be attributed to removal of the EPW.

## Discussion

Re-explorations for acute bleeding or haematoma evacuation are relatively frequent after major cardiac procedures, especially as modern guidelines recommend operating patients on aspirin or even on DAPT, for example in coronary surgery [4]. Reopening of a sternotomy potentially complicates wound healing, prolonging hospital stay and elevating costs [5]. Removal of the temporary EPW can potentially complicate the postoperative course of a cardiac surgical patient through acute bleeding and pericardial tamponade, nevertheless the exact incidence of this complication remains unclear, as there is a sparsity of data published in the literature.

Use of temporary epicardial pacemaker wires to manage possible complications, as bradycardia, postoperative atrial fibrillation with need for cardiac defibrillation or atrioventricular block, is a common procedure in cardiac surgery [1]. Although need of EPW can be questioned in selected cases, i.e. low-risk aortic valve replacement [6–8], presence of these wires can be very helpful in weaning from cardiopulmonary bypass or improving cardiac output in the early postoperative period [9].

At our department, EPW are sutured on the right atrium and right ventricle in every patient, except in presence of atrial fibrillation, where the atrial wire is omitted. We use a single type of temporary epicardial wires over the last years. Implantation of this type of wires does not necessitate any extra stitches.

The timing of EPW removal depends on the need for pacing postoperatively. These temporary wires are normally removed on the 4^th^ to 5^th^ POD by stable cardiac rhythm or later in cases where a cardioversion due to persistent postoperative atrial fibrillation is needed or a transient atrioventricular block demands pacing. Coagulation screening is made by every patient who is on VKA or high-dose unfractionated heparin before removing of these temporary wires, in order to prevent bleeding.

Several serious and somewhat bizarre complications after removal of EPW have been described in the literature [10–13]. Mahon et al. mention a less than 1% need for re-exploration due to pericardial tamponade after removal of EPW in a retrospective study involving more than 23000 patients with cardiac surgery [3]. These data coincide with our findings, showing eight patients with pericardial tamponade, where removal of EPW was the definite cause of bleeding (incidence 0.18%). Defining removal of the EPW as the cause of bleeding was somewhat arduous as an active bleeding from the insertion site of the wires was found and needed to be surgically managed in only two of the eight patients. In the remaining six patients, echocardiography was normal before removal of the wires, whereas the clinical symptoms directly after taking out the wires were characteristic of a pericardial tamponade. Whether a late removal of the EPW can result in pericardial bleeding, it remains totally unclear.

Our patients were managed either with re-exploration through repeat sternotomy or with the insertion of a subxiphoid pericardial drainage. Unfortunately, the outcome was fatal in two cases (2 of 8), whereas need for blood transfusion and wound infection due to resternotomy complicated three of the remaining 6 patients. These data support the finding that pericardial tamponade after removal of the EPW increases both morbidity and mortality.

In addition, our database revealed six more cases with a late re-exploration after the 14 POD. All of these patients had been discharged from the hospital without any evidence of a significant pericardial effusion in the echocardiographic study, with the EPW being removed after echocardiography. Three of these patients, which were on VKA, had a documented serious derailment of INR during their rehabilitation. By the re-exploration of these patients, no definite cause of bleeding could be verified.

After studying of these data, we modified the standard procedure of removal of the EPW at our department. In order to minimize the risk of serious bleeding, we initiate the oral anticoagulation regimen – VKA or NOAC – after the EPW have been taken out. Although none of the eight patients were on NOACs, we believe that it is prudent to commence these regimens after pulling out the EPW, as no standard screening for NOACs is routinely used [14]. On the other hand, patients who have a strong indication for DAPT, for example by coronary intervention with drug eluting stent implantation in the short-time perioperative period, still have their EPW removed under this medication. Whether it is rational to commence with the second antiplatelet agent after taking out the wires, it is dependent upon the indication for DAPT. Furthermore, we recommend that a high-dosed LMWH should be avoided 12 h before removal of EPW, and that intravenous administration of heparin should be paused for at least 3–4 h. Finally, performing the echocardiographic study after removal of the EPW, although a standard procedure by paediatric patients at our department is probably unrealistic as it is time-consuming and costly and even detection of some pericardial effusion may not prevent tamponade at a later time. Nevertheless, pre-removal echocardiography may detect a pericardial effusion that is clinically not apparent, but potentially aggravated by the EPW removal.

## Conclusion

We conclude, that a serious bleeding necessitating re-exploration of a patient after removal of the EPW is a very rare complication (under 0.2%), which could probably be avoided or further minimized by adjusting the antiplatelet or anticoagulant medication in the postoperative period at the time of removal of the wires. This complication increases both morbidity and mortality. The need of temporary pacing after cardiac surgery, although debatable by some surgeons for selected cases, remains a standard at most departments.

## Abbreviations

aPTT: Activated partial thromboplastin time
CABG: Coronary artery bypass grafting
DAPT: Dual antiplatelet therapy
EPW: Epicardial pacemaker wires
INR: International normalized ratio
LMWH: Low molecular weight heparin
NOAC: Novel oral anticoagulants
POD: Postoperative day
VKA: Vitamin K antagonist
